# P-2123. Unveiling Mortality Patterns in Candidemia: Insights from a Multi-Species Analysis from a Global Research Network

**DOI:** 10.1093/ofid/ofae631.2279

**Published:** 2025-01-29

**Authors:** George R Thompson, Soubhi Alhayek, Daniel B Chastain, Carolina Ferraz, Jorge Salinas, Stefan Sillau, Andrés F Henao Martínez

**Affiliations:** University of California Davis Medical Center, Sacramento, CA; University of California at Davis, Sacramento, California; University of Georgia College of Pharmacy, Albany, GA; University of Colorado School of Medicine - Anschutz Medical Campus, Sao Paulo, Sao Paulo, Brazil; Stanford University, Palo Alto, CA; University of Colorado, Denver, Colorado; University of Colorado Anschutz Medical Campus, Aurora, Colorado

## Abstract

**Background:**

Candidemia remains a critical concern in healthcare settings due to its high mortality rates and increasing prevalence of multidrug-resistant strains. Understanding the impact of different Candida species on patient outcomes is crucial for effective management and treatment strategies. This study aims to comprehensively analyze the association between Candida species and mortality in candidemia cases.

Percentages of 1-year mortality by Candida species.

**Figure:**
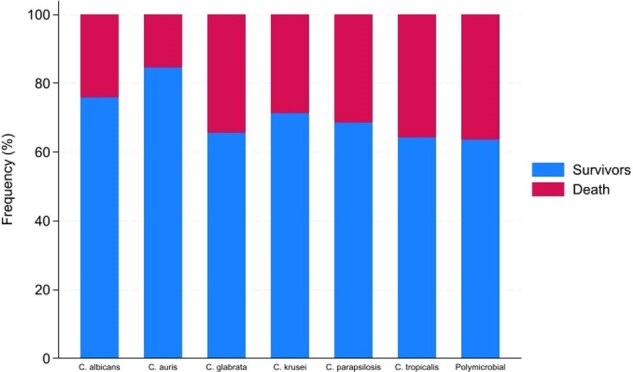

**Methods:**

We queried TriNetX, a global research network database (https://trinetx.com/), to identify patients with positive candidemia in blood by PCR testing from 2020-2023. We captured mortality as the primary outcome at one year in patients with candidemia categorized by Candida species. The time to death within the one year period was assessed using Kaplan-Meier plots, along with the non-parametric log-rank and Peto-Peto tests. Additionally, Cox proportional hazards (PH) models, both unadjusted and adjusted for demographic and comorbidity covariates, were employed for comparative analysis.

Unadjusted Kaplan-Mier Survival analysis with failure estimates in candidemia by Candida species

**Figure:**
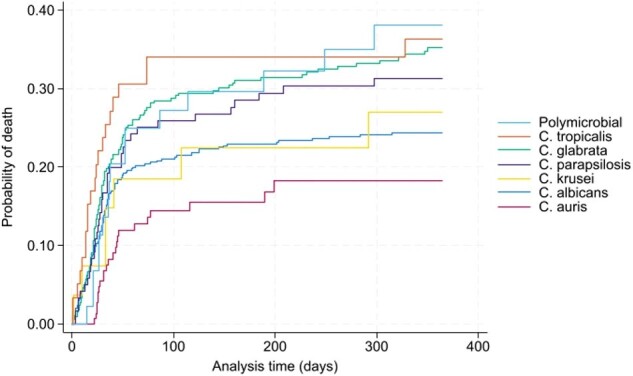

**Results:**

We captured 1,233 candidemia episodes during the study period. The distribution of Candida species was *C. albicans* (498, 40.4%), *C. glabrata* (333, 27%), *C. auris* (151, 12%), *C. parapsilosis* (121, 9.8%), *C. tropicalis* (59, 5%), *C. krusei* (28, 2%), and poly-candidemia (44, 4%) (Table 1). The one-year mortality varied across species, ranging from 15.3% to 36.6% (Figure 1). The unadjusted Kaplan-Mier Survival analysis showed that poly-candidemia, followed by *C. tropicalis,* had a worse survival than *C. auris,* which had the lowest risk (Figure 2). Adjusted Cox PH model found *C. albicans, C. glabrata, C. parapsilosis, C. tropicalis*, and poly-candidemia had statistically significantly higher mortality rates than *C. auris*. Additionally, age, non-Hispanic ethnicity, residence in the Western US region, and a higher Charlson comorbidity index value emerged as independent predictors of increased mortality (Table 2).

Clinical features by Candida species

**Figure:**
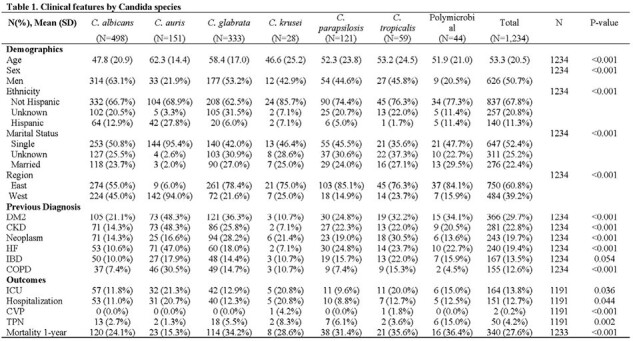

**Conclusion:**

Among patients with candidemia, we found an overall 1-year mortality of 28%. The associated resistance of *C. auris* may translate into a decreased virulence and a survival advantage. Additionally, older age and a higher comorbidity burden were associated with 1-year mortality.

**Figure:**
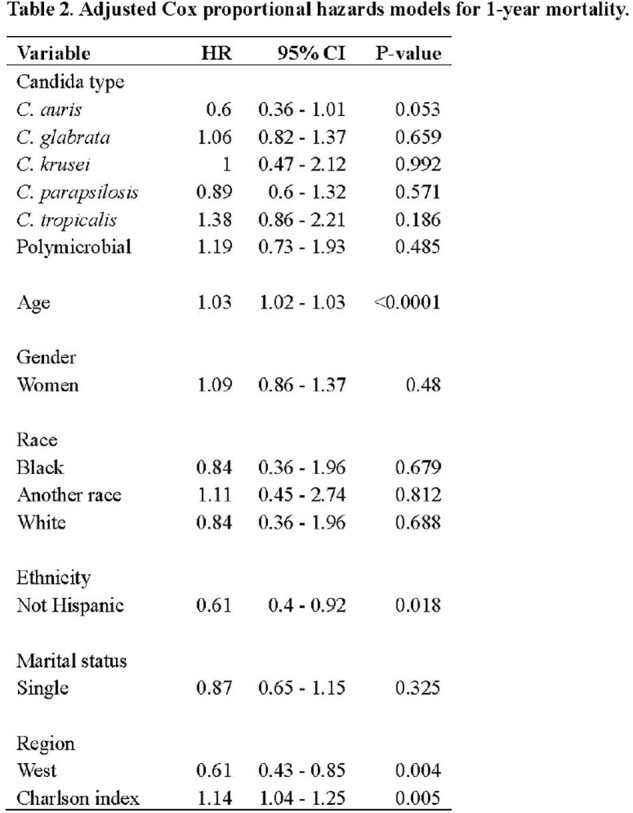

**Disclosures:**

George R. Thompson, III, MD, Astellas: Advisor/Consultant|Cidara: Advisor/Consultant|Cidara: Grant/Research Support|F2G: Advisor/Consultant|F2G: Grant/Research Support|Melinta: Advisor/Consultant|Melinta: Grant/Research Support|Mundipharma: Advisor/Consultant|Mundipharma: Grant/Research Support|Pfizer: Advisor/Consultant

